# Hemagglutinin Gene Clade 3C.2a Influenza A(H3N2) Viruses, Alachua County, Florida, USA, 2014–15

**DOI:** 10.3201/eid2201.151019

**Published:** 2016-01

**Authors:** John A. Lednicky, Nicole M. Iovine, Joe Brew, Julia C. Loeb, Jonathan D. Sugimoto, Kenneth H. Rand, J. Glenn Morris

**Affiliations:** University of Florida, Gainesville, Florida, USA (J.A. Lednicky, N.M. Iovine, J. Brew, J.C. Loeb, K.H. Rand, J.G. Morris, Jr.);; Florida Department of Health, Gainesville (J. Brew);; Fred Hutchinson Cancer Research Center, Seattle, Washington, USA (J.D. Sugimoto)

**Keywords:** influenza, viruses, Florida, hemagglutinin, vaccines, influenza A(H3N2), respiratory infections

## Abstract

Influenza A(H3N2) strains isolated during 2014–15 in Alachua County, Florida, USA, belonged to hemagglutinin gene clade 3C.2a. High rates of influenza-like illness and confirmed influenza cases in children were associated with a decrease in estimated vaccine effectiveness. Illnesses were milder than in 2013–14; severe cases were concentrated in elderly patients with underlying diseases.

Influenza vaccines are less effective when antigenic drift occurs (i.e., when amino acids change at the antigenic epitopes of the viruses) (*1*,*2*). The influenza A(H3N2) virus for the 2014–15 vaccine was an A/Texas/50/2012 (H3N2)–like virus, which is a hemagglutinin (HA) gene clade 3C.1 virus (*3*–*5*). We identified an HA gene clade 3C.2a virus as the predominant strain in Gainesville, Florida, USA, during the 2014–15 influenza season and assessed vaccine effectiveness and clinical outcomes associated with this strain. All work was approved by the University of Florida (Gainesville) Institutional Review Board.

## The Study

Influenza in Gainesville and the surrounding Alachua County began in September 2014. Multiplex PCR (eSensor respiratory viral panel; GenMark Dx, Carlsbad, CA, USA) for patients seen at a major teaching hospital in Alachua County indicated that >95% of illnesses were caused by H3N2 strains. Influenza A(H3N2) virus HA, neuraminidase (NA), and matrix (M) gene sequences from 5 specimens collected from patients in November 2014 were directly sequenced from the specimens, and corresponding first-passage viruses were isolated in MDCK cells by using previously described primers (*6*,*7*). These viruses were designated as A/Gainesville (H3N2) isolates 5–9 from 2014 (e.g., A/GNVL/05/2014) (Table 1). For all, the consensus viral genomic sequences determined directly from clinical specimens and their matched virus isolates were identical.

**Table 1:** Amino acid sequences at hemagglutinin major immunogenic epitopes A and B1 of influenza A(H3N2) viruses, Gainesville, Florida, USA, November 2014

Strain	Epitope A, aa 121–146																											Epitope B1, aa 155–163								
Consensus sequence*	**N**	**N**	**E**	**S**	**F**	**N**	**W**	**T**	**G**	**V**	**T**	**Q**	**N**	**G**	**T**	**S**	**S**	**A**	**C**	**K**	**R**	**R**	**S**	**N**	**N**	**S**	**T**	**H**	**L**	**K**	**F**	**K**	**Y**	**P**	**A**
A/Texas/ 50/2012†								**N**												**I**											**N**					
A/GNVL/ 01/2014‡								**A**												**I**		**G**			**S**						**N**					
A/GNVL/ 05/2014§																				**I**				**S**	**S**						**N**	**Y**	**T**			
A/GNVL/ 06/2014§																				**I**				**S**	**S**						**N**	**Y**	**T**			
A/GNVL/ 07/2014§																				**I**				**S**	**S**						**N**	**Y**	**T**			
A/GNVL/ 08/2014§																				**I**				**S**	**S**						**N**	**Y**	**T**			
A/GNVL/ 09/2014¶																				**I**				**S**	**S**						**N**	**Y**	**T**			

*Yamashita et al. (*9*). †Virus in 2013–14 and 2014–15 vaccines, Northern Hemisphere; GenBank accession no. KC892952.1. ‡GenBank accession no. KJ439217. §Viruses in nasopharyngeal swabs collected in November 2014. ¶Virus in sputum collected in November 2014.

The deduced HA major antigenic epitopes A and B1 of the 5 viruses isolated had some amino acids not found in the HA protein of vaccine strain A/Texas/50/2012 (H3N2) (Table 1). No changes were observed for major antigenic epitopes B2, C1, C2, D, and E. Changes at the major antigenic epitopes included N144S, N145S, F159Y, K160T, N225D, and at other epitopes L3I and Q311H; those changes are characteristic of HA gene clade 3C.2a viruses (*4*). Substitutions at residues 144 and 160 have been associated with the loss and the gain, respectively, of possible N-linked glycosylation sites of the HA protein (*8*,*9*). An H3N2 virus isolated in February 2014 in Gainesville, designated A/GNVL/01/2014 (H3N2), was from a different HA gene clade (Table 1), suggesting that the clade 3C.2a viruses were introduced locally later. The NA proteins of the 5 viruses from November 2014 also differed from those of the Northern Hemisphere H3N2 vaccine strain at 3 amino acids, whereas the M proteins were identical.

Data on frequency of medically attended influenza-like illness (ILI) for the 2014–15 influenza season were obtained from Florida’s Electronic Surveillance System for the Early Notification of Community-based Epidemics (ESSENCE) for Alachua County; the 13-county Florida Department of Health Region 3 (2010 population: 2.2 million), which includes Alachua County; and the state of Florida (*10*,*11*). ILI-associated emergency department (ED) visits were defined as visits for which a chief complaint contained the word “influenza” or “flu” or the word “fever” plus “cough” and/or “sore throat.” Region 3 and Florida are comparison areas, so they exclude Alachua County data. For the 2014–15 influenza season (September 28, 2014–May 23, 2015), the rate of reported ILI for Alachua County residents was 499 cases per 100,000 population. Sixteen percent of cases were among children <17 years of age, a significant increase over 2013–14, when 11% of cases were among children in this age group (p = 0.001, Pearson χ^2^ test, 2-tailed). The pattern for laboratory-confirmed influenza cases in our hospital ED was similar; although the rate of confirmed influenza cases among adult ED patients did not increase, the rate of confirmed cases among pediatric ED patients increased significantly from 6/1,000 unique ED patients in 2013–14 to 43/1,000 in 2014–15 (p<0.0001, χ^2^).

Alachua County has a nationally recognized school-based influenza vaccination program that administers live attenuated influenza vaccine (FluMist; AstraZeneca, Wilmington, DE, USA); parenteral influenza vaccine is administered by private physicians (*12*). Since 2010, the average overall influenza vaccination rate for children 0–17 years of age in Alachua County has been approximately 45%. As previously reported (*11*), this rate has correlated with a reduction in rates of ED visits for ILI (as reported through the Florida ESSENCE surveillance system) among children in Alachua County, compared with those in Florida Region 3 and the state of Florida. Corresponding estimates of vaccine effectiveness (relative reductions in risk) in Alachua County, by year, are shown in Table 2. Accompanying the increase in cases among children for 2014–15 was a small but significant decrease in estimated effectiveness of vaccination for children in Florida Region 3 (Table 2).

**Table 2:** Estimated influenza vaccine effectiveness for children in Alachua County, Florida, USA, as compared with Florida Region 3, and Florida as a whole*

Age group, y	Influenza season	Region 3, % (95% CI)	Florida, % (95% CI)
5–17	2012–13	71 (67–76)	73 (68–78)
	2013–14	73 (68–79)	77 (72–82)
	2014–15	63 (58–69)	73 (68–78)
0–4	2012–13	82 (79–86)	84 (80–87)
	2013–14	85 (82–89)	88 (85–91)
	2014–15	80 (77–84)	86 (83–89)

*Estimates are based on reports of influenza-like illness from emergency departments reported through Florida’s Electronic Surveillance System for the Early Notification of Community-based Epidemics. See Tran et al. (*11*) for detailed methods. Region 3 and Florida are comparison areas, so they exclude Alachua County data.

As a marker of disease severity, we identified the number of patients at our hospital admitted to the medical intensive care unit (MICU) because of influenza by querying the University of Florida Health Integrated Data Repository for patients with International Classification of Diseases, Ninth Revision, diagnostic codes for influenza (487) or novel influenza (488) listed in the first 10 diagnoses. In the 2014–15 influenza season, 1.8% of MICU patients carried the diagnosis of influenza, a significant reduction from the 4.8% of MICU admissions in 2013–14, when an H1N1 dominated (p<0.0001, Fisher exact test, 2-tailed). The mean age for persons admitted to MICU with influenza in 2014–15 was 60 years, compared with 50 years for 2013–14 (p = 0.006, Mann-Whitney U test, 2-tailed). Seven of these MICU patients died; all were >50 years of age (mean 70 years). Patient ages were significantly higher than for persons with fatal cases in 2013–14 (mean 48 years) (p = 0.04, Mann-Whitney U test, 2-tailed), with a significantly higher Charleston co-morbidity index (4.9 vs. 2.4, p = 0.04, Mann-Whitney U test, 2-tailed).

## Conclusions

The predominant influenza strain in this community in 2013–14 was an H1N1 variant bearing the D225G polymorphism, which was associated with more severe disease outside of elderly age groups (*10*). Rates of illness severe enough to warrant MICU admission with the H3N2 clade 3C.2a strain circulating in 2014–15 were significantly lower than those with H1N1 in 2013–14 and occurred in a significantly older age group with a higher rate of co-morbidities, more closely matched observations from in the 2012–13 H3N2 influenza season. These findings highlight the substantial strain-related differences in virulence that occur from year to year and the importance of ongoing genetic monitoring of strains circulating within communities.

We noted an increase in influenza incidence in children <17 years of age in Alachua County during 2014–15. This increase was probably due, at least in part, to a decrease in vaccine effectiveness with the clade 3C.2a strain in this highly vaccinated population of children. 

Our hospital-based ED and MICU data use laboratory-confirmed cases, whereas the regional and state data and vaccine effectiveness calculations are based on ILI data from the Florida ESSENCE system, which are potentially less reliable. Although we found data trends in the 2 systems to be consistent, our findings underscore the need for expanded surveillance of laboratory-confirmed cases statewide, particularly with the increasingly widespread distribution of clade 3C.2a strains in the Americas and Europe (*13*). These issues might be particularly relevant in light of the recent World Health Organization recommendation to substitute the HA gene clade 3C.3a virus A/Switzerland/9715293/2013 (H3N2) (rather than a clade 3C.2a virus) for A/Texas/50/2012 (H3N2) in the 2015 vaccine for the Northern Hemisphere.
